# Biphenyl-2,4,4′,6-tetra­carb­oxy­lic acid monohydrate

**DOI:** 10.1107/S1600536813019624

**Published:** 2013-08-03

**Authors:** Ye-Nan Wang, Jun Zhao

**Affiliations:** a College of Mechanical and Material Engineering, China Three Gorges University, Yichang 443002’ , People’s Republic of China

## Abstract

In the title compound, C_16_H_10_O_8_·H_2_O, the dihedral angle between the benzene rings is 71.59 (8)°. The COOH groups make dihedral angles of 10.3 (2), 30.8 (2), 11.3 (2) and 42.3 (2)° with their attached rings. In the crystal, O—H⋯O hydrogen bonds link the components forming a three-dimensional supra­molecular network.

## Related literature

For general background to the use of aromatic carboxyl­ates as building blocks for the construction of various architectures, see: Yaghi *et al.* (2003 ▶); Zhao *et al.* (2012 ▶).
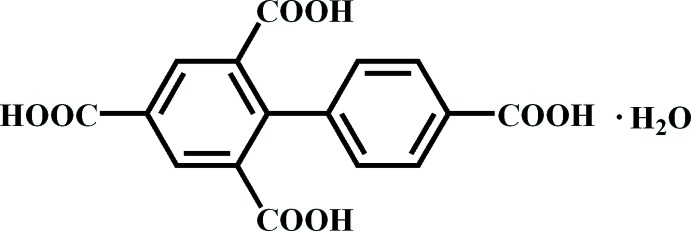


## Experimental

### 

#### Crystal data

C_16_H_10_O_8_·H_2_O
*M* *_r_* = 348.26
Monoclinic, 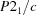

*a* = 5.638 (4) Å
*b* = 16.160 (11) Å
*c* = 16.798 (12) Å
β = 92.524 (12)°
*V* = 1528.9 (19) Å^3^
*Z* = 4
Mo *K*α radiation
μ = 0.13 mm^−1^
*T* = 296 K
0.21 × 0.18 × 0.17 mm

#### Data collection

Bruker SMART CCD diffractometer
Absorption correction: multi-scan (*SADABS*; Sheldrick, 1996 ▶) *T*_min_ = 0.974, *T*_max_ = 0.979
15936 measured reflections
3516 independent reflections
3070 reflections with *I* > 2σ(*I*)
*R*_int_ = 0.124

#### Refinement

*R*[*F* ^2^ > 2σ(*F* ^2^)] = 0.052
*wR*(*F* ^2^) = 0.152
*S* = 1.02
3516 reflections
232 parameters
3 restraints
H atoms treated by a mixture of independent and constrained refinement
Δρ_max_ = 0.30 e Å^−3^
Δρ_min_ = −0.26 e Å^−3^

### 

Data collection: *SMART* (Bruker, 2007 ▶); cell refinement: *SAINT* (Bruker, 2007 ▶); data reduction: *SAINT*; program(s) used to solve structure: *SHELXS97* (Sheldrick, 2008 ▶); program(s) used to refine structure: *SHELXL97* (Sheldrick, 2008 ▶); molecular graphics: *SHELXTL* (Sheldrick, 2008 ▶); software used to prepare material for publication: *SHELXTL*.

## Supplementary Material

Crystal structure: contains datablock(s) I, global. DOI: 10.1107/S1600536813019624/lh5634sup1.cif

Structure factors: contains datablock(s) I. DOI: 10.1107/S1600536813019624/lh5634Isup2.hkl

Supplementary material file. DOI: 10.1107/S1600536813019624/lh5634Isup3.cml

Additional supplementary materials:  crystallographic information; 3D view; checkCIF report

## Figures and Tables

**Table 1:** Hydrogen-bond geometry (Å, °)

*D*—H⋯*A*	*D*—H	H⋯*A*	*D*⋯*A*	*D*—H⋯*A*
O2—H2*A*⋯O3^i^	0.82	1.87	2.688 (2)	179
O4—H4*B*⋯O1*W* ^ii^	0.82	1.91	2.725 (2)	177
O6—H6*B*⋯O8^iii^	0.82	1.82	2.636 (2)	178
O7—H7*B*⋯O1*W* ^iv^	0.82	1.87	2.688 (2)	176
O1*W*—H1*WB*⋯O1	0.85 (1)	2.09 (1)	2.818 (2)	143 (2)
O1*W*—H1*WA*⋯O5^v^	0.86 (1)	1.98 (1)	2.787 (2)	157 (2)

Symmetry codes: (i) 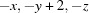; (ii) 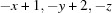; (iii) 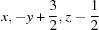; (iv) 
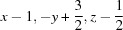
; (v) 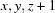.

**Fig. 1.**
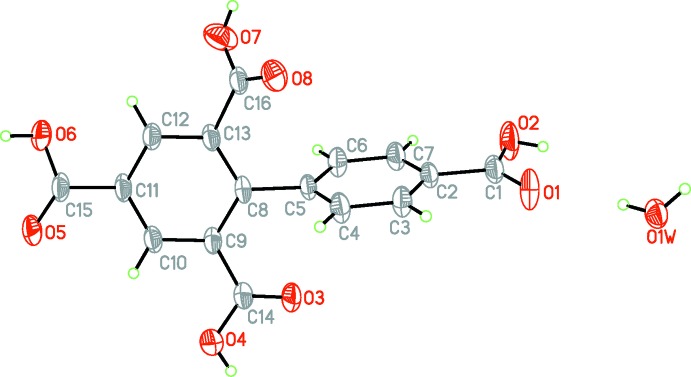
The molecular structure of the title compound with displacement ellipsoids at the 50% probability level.

**Fig. 2.**
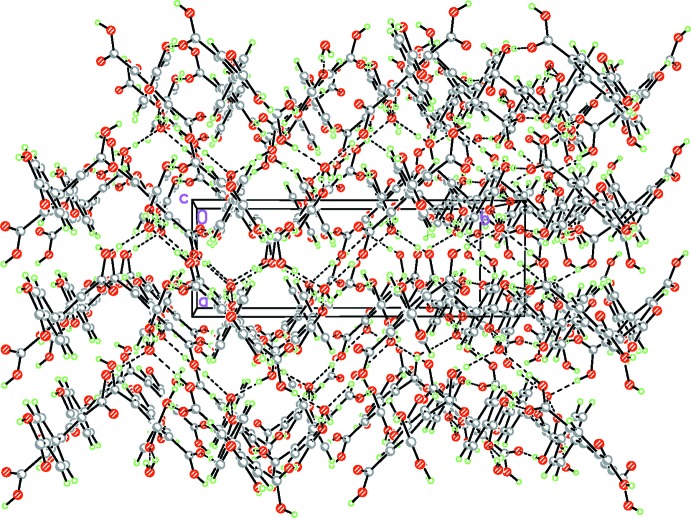
Part of the crystal structure with hydrogen bonds shown as dashed lines.

## supplementary crystallographic information

### 1. Comment

Aromatic carboxylates have been proven to be effective building blocks for the design and construction of coordination polymers exhibiting remarkable polymeric structural motifs due to their rich coordination modes (Yaghi *et al*.,2003; Zhao *et al.*, 2012). Recently, we attempted to synthesize an Sm^III^ complex with the ligand in hydrothermal synthesis conditions. However the title organic salt was obtained, its structure is reported here.

The molecular structure of the title compound is shown in Fig. 1. The asymmetric unit comprises one bipheny1,2,4,4'6-tetracarboxylic acid and one solvent water molecule. The dihedral angle between the two benzene rings of the bipheny-1,2,4,4'6-tetracarboxylic acid is 71.59 (8)°. This may be the result of intermolecular O—H···O interactions and steric effects. In the crystal, extensive O—H···O hydrogen bonds invoving bipheny-1,2,4,4'6-tetracarboxylic acid molecules and water molecules link the components into a three-dimensional supramolecular network (Fig. 2).

### 2. Experimental

All chemicals were of reagent grade quality obtained from commercial sources and used without further purification. A mixture of bipheny1,2,4,4'6-tetracarboxylic acid (0.0370 g, 0.1 mmol), Sm(NO_3_)_3_.6H_2_O (0.0444 g, 0.1 mmol) and water (12 ml) were placed in a 23 ml Teflon-lined stainless steel reactor and heated at 393 K for 2 days, and then cooled to room temperature at 10 K h^-1^ to obtain colorless prism-shaped crystals suitable for X-ray analysis.

### 3. Refinement

The H atoms bonded to C and carboxylate O atoms were positioned geometrically (C—H = 0.93, O—H = 0.82 Å) and allowed to ride on their parent atoms, with *U*_iso_(H) value equal to 1.2*U*_eq_(C) or 1.5*U*_eq_(O). The H atoms bonded to water were located in a difference Fourier map and refined with O—H distance restraint of 0.85±0.01 Å, *U*_iso_(H) = 1.5*U*_eq_(O).

### Crystal data

**Table:**

C_16_H_10_O_8_·H_2_O	*F*(000) = 720
*M**_r_* = 348.26	*D*_x_ = 1.513 Mg m^−^^3^
Monoclinic, *P*2_1_/*c*	Mo *K*α radiation, λ = 0.71073 Å
Hall symbol: -p 2ybc	Cell parameters from 4298 reflections
*a* = 5.638 (4) Å	θ = 3.5–27.6°
*b* = 16.160 (11) Å	µ = 0.13 mm^−^^1^
*c* = 16.798 (12) Å	*T* = 296 K
β = 92.524 (12)°	Prism, colorless
*V* = 1528.9 (19) Å^3^	0.21 × 0.18 × 0.17 mm
*Z* = 4	

### Data collection

**Table:**

Bruker SMART CCD diffractometer	3516 independent reflections
Radiation source: fine-focus sealed tube	3070 reflections with *I* > 2σ(*I*)
Graphite monochromator	*R*_int_ = 0.124
φ and ω scans	θ_max_ = 27.6°, θ_min_ = 3.5°
Absorption correction: multi-scan (*SADABS*; Sheldrick, 1996)	*h* = −7→7
*T*_min_ = 0.974, *T*_max_ = 0.979	*k* = −20→20
15936 measured reflections	*l* = −21→21

### Refinement

**Table:**

Refinement on *F*^2^	Primary atom site location: structure-invariant direct methods
Least-squares matrix: full	Secondary atom site location: difference Fourier map
*R*[*F*^2^ > 2σ(*F*^2^)] = 0.052	Hydrogen site location: inferred from neighbouring sites
*wR*(*F*^2^) = 0.152	H atoms treated by a mixture of independent and constrained refinement
*S* = 1.02	*w* = 1/[σ^2^(*F*_o_^2^) + (0.0821*P*)^2^ + 0.3015*P*] where *P* = (*F*_o_^2^ + 2*F*_c_^2^)/3
3516 reflections	(Δ/σ)_max_ < 0.001
232 parameters	Δρ_max_ = 0.30 e Å^−^^3^
3 restraints	Δρ_min_ = −0.26 e Å^−^^3^

### Special details

**Table:**

Geometry. All e.s.d.'s (except the e.s.d. in the dihedral angle between two l.s. planes) are estimated using the full covariance matrix. The cell e.s.d.'s are taken into account individually in the estimation of e.s.d.'s in distances, angles and torsion angles; correlations between e.s.d.'s in cell parameters are only used when they are defined by crystal symmetry. An approximate (isotropic) treatment of cell e.s.d.'s is used for estimating e.s.d.'s involving l.s. planes.
Refinement. Refinement of *F*^2^ against ALL reflections. The weighted *R*-factor *wR* and goodness of fit *S* are based on *F*^2^, conventional *R*-factors *R* are based on *F*, with *F* set to zero for negative *F*^2^. The threshold expression of *F*^2^ > σ(*F*^2^) is used only for calculating *R*-factors(gt) *etc*. and is not relevant to the choice of reflections for refinement. *R*-factors based on *F*^2^ are statistically about twice as large as those based on *F*, and *R*- factors based on ALL data will be even larger.

### Fractional atomic coordinates and isotropic or equivalent isotropic displacement parameters (Å^2^)

**Table:**

	*x*	*y*	*z*	*U*_iso_*/*U*_eq_	
C1	−0.0695 (3)	0.89761 (9)	0.06051 (8)	0.0368 (3)	
C2	−0.0558 (3)	0.89091 (9)	−0.02802 (8)	0.0319 (3)	
C3	0.1413 (3)	0.85407 (10)	−0.06078 (8)	0.0369 (3)	
H3A	0.2633	0.8333	−0.0274	0.044*	
C4	0.1565 (3)	0.84821 (10)	−0.14271 (8)	0.0368 (3)	
H4A	0.2885	0.8236	−0.1641	0.044*	
C5	−0.0247 (2)	0.87892 (8)	−0.19285 (7)	0.0298 (3)	
C6	−0.2205 (3)	0.91575 (10)	−0.15985 (8)	0.0367 (3)	
H6A	−0.3424	0.9365	−0.1932	0.044*	
C7	−0.2365 (3)	0.92190 (10)	−0.07773 (8)	0.0366 (3)	
H7A	−0.3682	0.9468	−0.0563	0.044*	
C8	−0.0256 (2)	0.87106 (8)	−0.28226 (7)	0.0291 (3)	
C9	0.1157 (2)	0.92037 (8)	−0.33066 (7)	0.0303 (3)	
C10	0.1001 (3)	0.91203 (9)	−0.41364 (8)	0.0336 (3)	
H10A	0.2018	0.9420	−0.4448	0.040*	
C11	−0.0664 (3)	0.85925 (9)	−0.44958 (7)	0.0336 (3)	
C12	−0.2109 (3)	0.81166 (9)	−0.40302 (8)	0.0332 (3)	
H12A	−0.3254	0.7773	−0.4270	0.040*	
C13	−0.1844 (2)	0.81537 (8)	−0.32018 (7)	0.0305 (3)	
C14	0.2767 (2)	0.98523 (9)	−0.29572 (8)	0.0316 (3)	
C15	−0.0870 (3)	0.85389 (10)	−0.53843 (8)	0.0375 (3)	
C16	−0.3229 (3)	0.75439 (9)	−0.27373 (8)	0.0325 (3)	
O1W	0.24574 (18)	0.88370 (7)	0.26856 (6)	0.0378 (3)	
H1WB	0.159 (3)	0.8987 (13)	0.2288 (8)	0.057*	
O1	0.0881 (3)	0.87787 (9)	0.10711 (6)	0.0544 (4)	
O2	−0.2753 (2)	0.92774 (10)	0.08217 (7)	0.0603 (4)	
H2A	−0.2625	0.9434	0.1286	0.090*	
O3	0.2393 (2)	1.02125 (8)	−0.23453 (6)	0.0467 (3)	
O4	0.4623 (2)	1.00041 (8)	−0.33852 (7)	0.0479 (3)	
H4B	0.5466	1.0353	−0.3159	0.072*	
O5	0.0525 (3)	0.88594 (9)	−0.58157 (6)	0.0556 (4)	
O6	−0.2751 (3)	0.81178 (9)	−0.56371 (6)	0.0564 (4)	
H6B	−0.2663	0.8019	−0.6114	0.085*	
O7	−0.5391 (2)	0.74324 (9)	−0.30234 (8)	0.0545 (4)	
H7B	−0.5991	0.7040	−0.2796	0.082*	
O8	−0.2375 (2)	0.71750 (8)	−0.21700 (7)	0.0511 (3)	
H1WA	0.158 (3)	0.8750 (16)	0.3080 (9)	0.077*	

### Atomic displacement parameters (Å^2^)

**Table:**

	*U*^11^	*U*^22^	*U*^33^	*U*^12^	*U*^13^	*U*^23^
C1	0.0543 (8)	0.0375 (7)	0.0190 (6)	−0.0055 (6)	0.0060 (5)	−0.0034 (5)
C2	0.0443 (7)	0.0347 (7)	0.0171 (6)	−0.0052 (6)	0.0058 (5)	−0.0024 (5)
C3	0.0435 (7)	0.0483 (8)	0.0189 (6)	0.0039 (6)	0.0000 (5)	−0.0002 (5)
C4	0.0403 (7)	0.0500 (8)	0.0205 (6)	0.0055 (6)	0.0056 (5)	−0.0026 (6)
C5	0.0403 (7)	0.0347 (7)	0.0147 (6)	−0.0033 (5)	0.0041 (5)	−0.0009 (5)
C6	0.0408 (7)	0.0492 (8)	0.0201 (6)	0.0070 (6)	0.0010 (5)	−0.0016 (5)
C7	0.0421 (7)	0.0463 (8)	0.0219 (6)	0.0034 (6)	0.0065 (5)	−0.0053 (5)
C8	0.0387 (6)	0.0347 (7)	0.0142 (5)	0.0040 (5)	0.0038 (5)	−0.0009 (5)
C9	0.0413 (7)	0.0324 (7)	0.0173 (6)	0.0012 (5)	0.0031 (5)	−0.0014 (5)
C10	0.0469 (7)	0.0364 (7)	0.0179 (6)	−0.0033 (6)	0.0069 (5)	−0.0003 (5)
C11	0.0519 (8)	0.0349 (7)	0.0144 (6)	−0.0012 (6)	0.0042 (5)	−0.0004 (5)
C12	0.0479 (7)	0.0346 (7)	0.0173 (6)	−0.0039 (6)	0.0019 (5)	−0.0018 (5)
C13	0.0423 (7)	0.0328 (7)	0.0167 (6)	0.0003 (5)	0.0048 (5)	0.0009 (5)
C14	0.0396 (7)	0.0357 (7)	0.0196 (6)	0.0014 (5)	0.0019 (5)	0.0008 (5)
C15	0.0583 (9)	0.0386 (8)	0.0157 (6)	−0.0041 (6)	0.0040 (5)	−0.0024 (5)
C16	0.0468 (7)	0.0336 (7)	0.0177 (6)	−0.0005 (6)	0.0059 (5)	−0.0008 (5)
O1W	0.0384 (5)	0.0507 (6)	0.0245 (5)	0.0005 (4)	0.0056 (4)	−0.0009 (4)
O1	0.0762 (8)	0.0685 (9)	0.0182 (5)	0.0146 (7)	−0.0019 (5)	−0.0023 (5)
O2	0.0637 (7)	0.0971 (11)	0.0207 (5)	0.0110 (7)	0.0100 (5)	−0.0130 (6)
O3	0.0637 (7)	0.0513 (7)	0.0259 (5)	−0.0128 (5)	0.0106 (5)	−0.0143 (4)
O4	0.0489 (6)	0.0598 (7)	0.0362 (6)	−0.0160 (5)	0.0131 (5)	−0.0146 (5)
O5	0.0808 (9)	0.0689 (8)	0.0179 (5)	−0.0253 (7)	0.0126 (5)	−0.0035 (5)
O6	0.0769 (9)	0.0744 (9)	0.0178 (5)	−0.0256 (7)	0.0019 (5)	−0.0063 (5)
O7	0.0483 (6)	0.0621 (8)	0.0528 (8)	−0.0122 (6)	−0.0002 (5)	0.0241 (6)
O8	0.0701 (8)	0.0563 (7)	0.0265 (5)	−0.0137 (6)	−0.0031 (5)	0.0156 (5)

### Geometric parameters (Å, º)

**Table:**

C1—O1	1.201 (2)	C10—H10A	0.9300
C1—O2	1.324 (2)	C11—C12	1.387 (2)
C1—C2	1.496 (2)	C11—C15	1.494 (2)
C2—C7	1.382 (2)	C12—C13	1.394 (2)
C2—C3	1.395 (2)	C12—H12A	0.9300
C3—C4	1.386 (2)	C13—C16	1.498 (2)
C3—H3A	0.9300	C14—O3	1.2079 (19)
C4—C5	1.387 (2)	C14—O4	1.3181 (19)
C4—H4A	0.9300	C15—O5	1.210 (2)
C5—C6	1.391 (2)	C15—O6	1.314 (2)
C5—C8	1.507 (2)	C16—O8	1.2061 (19)
C6—C7	1.390 (2)	C16—O7	1.303 (2)
C6—H6A	0.9300	O1W—H1WB	0.845 (9)
C7—H7A	0.9300	O1W—H1WA	0.856 (9)
C8—C13	1.403 (2)	O2—H2A	0.8200
C8—C9	1.4099 (19)	O4—H4B	0.8200
C9—C10	1.399 (2)	O6—H6B	0.8200
C9—C14	1.490 (2)	O7—H7B	0.8200
C10—C11	1.387 (2)		
			
O1—C1—O2	123.42 (14)	C11—C10—C9	120.31 (13)
O1—C1—C2	124.02 (15)	C11—C10—H10A	119.8
O2—C1—C2	112.56 (13)	C9—C10—H10A	119.8
C7—C2—C3	119.67 (13)	C12—C11—C10	119.92 (13)
C7—C2—C1	120.35 (13)	C12—C11—C15	120.62 (13)
C3—C2—C1	119.97 (13)	C10—C11—C15	119.46 (13)
C4—C3—C2	120.40 (13)	C11—C12—C13	119.94 (13)
C4—C3—H3A	119.8	C11—C12—H12A	120.0
C2—C3—H3A	119.8	C13—C12—H12A	120.0
C3—C4—C5	120.16 (13)	C12—C13—C8	121.27 (12)
C3—C4—H4A	119.9	C12—C13—C16	117.14 (12)
C5—C4—H4A	119.9	C8—C13—C16	121.51 (12)
C4—C5—C6	119.18 (13)	O3—C14—O4	123.16 (14)
C4—C5—C8	123.02 (12)	O3—C14—C9	123.23 (13)
C6—C5—C8	117.74 (12)	O4—C14—C9	113.60 (12)
C7—C6—C5	120.91 (13)	O5—C15—O6	124.40 (14)
C7—C6—H6A	119.5	O5—C15—C11	123.34 (14)
C5—C6—H6A	119.5	O6—C15—C11	112.26 (13)
C2—C7—C6	119.68 (13)	O8—C16—O7	123.88 (14)
C2—C7—H7A	120.2	O8—C16—C13	122.53 (14)
C6—C7—H7A	120.2	O7—C16—C13	113.54 (12)
C13—C8—C9	117.81 (12)	H1WB—O1W—H1WA	108.9 (14)
C13—C8—C5	118.72 (12)	C1—O2—H2A	109.5
C9—C8—C5	123.37 (12)	C14—O4—H4B	109.5
C10—C9—C8	120.50 (13)	C15—O6—H6B	109.5
C10—C9—C14	118.09 (12)	C16—O7—H7B	109.5
C8—C9—C14	121.35 (12)		
			
O1—C1—C2—C7	−174.12 (16)	C14—C9—C10—C11	−172.69 (13)
O2—C1—C2—C7	5.9 (2)	C9—C10—C11—C12	−2.9 (2)
O1—C1—C2—C3	5.1 (2)	C9—C10—C11—C15	177.47 (13)
O2—C1—C2—C3	−174.80 (15)	C10—C11—C12—C13	−1.6 (2)
C7—C2—C3—C4	−0.2 (2)	C15—C11—C12—C13	177.95 (13)
C1—C2—C3—C4	−179.45 (14)	C11—C12—C13—C8	4.8 (2)
C2—C3—C4—C5	−0.1 (2)	C11—C12—C13—C16	−172.06 (13)
C3—C4—C5—C6	0.2 (2)	C9—C8—C13—C12	−3.2 (2)
C3—C4—C5—C8	−177.01 (14)	C5—C8—C13—C12	173.20 (13)
C4—C5—C6—C7	−0.1 (2)	C9—C8—C13—C16	173.50 (12)
C8—C5—C6—C7	177.24 (13)	C5—C8—C13—C16	−10.09 (19)
C3—C2—C7—C6	0.3 (2)	C10—C9—C14—O3	148.67 (15)
C1—C2—C7—C6	179.54 (14)	C8—C9—C14—O3	−28.5 (2)
C5—C6—C7—C2	−0.1 (2)	C10—C9—C14—O4	−30.44 (18)
C4—C5—C8—C13	107.63 (17)	C8—C9—C14—O4	152.42 (13)
C6—C5—C8—C13	−69.63 (18)	C12—C11—C15—O5	−170.64 (17)
C4—C5—C8—C9	−76.17 (19)	C10—C11—C15—O5	9.0 (2)
C6—C5—C8—C9	106.56 (17)	C12—C11—C15—O6	10.4 (2)
C13—C8—C9—C10	−1.40 (19)	C10—C11—C15—O6	−170.01 (15)
C5—C8—C9—C10	−177.63 (12)	C12—C13—C16—O8	136.42 (16)
C13—C8—C9—C14	175.67 (12)	C8—C13—C16—O8	−40.4 (2)
C5—C8—C9—C14	−0.6 (2)	C12—C13—C16—O7	−41.20 (19)
C8—C9—C10—C11	4.5 (2)	C8—C13—C16—O7	141.96 (15)

### Hydrogen-bond geometry (Å, º)

**Table:**

*D*—H···*A*	*D*—H	H···*A*	*D*···*A*	*D*—H···*A*
O2—H2*A*···O3^i^	0.82	1.87	2.688 (2)	179
O4—H4*B*···O1*W*^ii^	0.82	1.91	2.725 (2)	177
O6—H6*B*···O8^iii^	0.82	1.82	2.636 (2)	178
O7—H7*B*···O1*W*^iv^	0.82	1.87	2.688 (2)	176
O1*W*—H1*WB*···O1	0.85 (1)	2.09 (1)	2.818 (2)	143 (2)
O1*W*—H1*WA*···O5^v^	0.86 (1)	1.98 (1)	2.787 (2)	157 (2)

Symmetry codes: (i) −*x*, −*y*+2, −*z*; (ii) −*x*+1, −*y*+2, −*z*; (iii) *x*, −*y*+3/2, *z*−1/2; (iv) *x*−1, −*y*+3/2, *z*−1/2; (v) *x*, *y*, *z*+1.

## References

[bb1] Bruker (2007). *SMART* and *SAINT* Bruker AXS Inc., Madison, Wisconsin, USA.

[bb2] Sheldrick, G. M. (1996). *SADABS* University of Göttingen, Germany.

[bb3] Sheldrick, G. M. (2008). *Acta Cryst.* A**64**, 112–122. 10.1107/S0108767307043930 18156677

[bb4] Yaghi, O. M., O’Keeffe, M., Ockwing, N. W., Chae, H. K., Eddaoudi, M. & Kim, J. (2003). *Nature*, **423**, 705–714. 10.1038/nature01650 12802325

[bb5] Zhao, J., Li, D. S., Ke, X. J., Liu, B., Zou, K. & Hu, H. M. (2012). *Dalton Trans.* **41**, 2560–2563. 10.1039/c2dt12170k 22223134

